# Selective targeting of glioma via the SCARB2 receptor: transcriptomic, proteomic and *in vitro* functional validation for Enterovirus A71 virotherapy

**DOI:** 10.3389/fcimb.2025.1709002

**Published:** 2025-10-23

**Authors:** Jinchuan Li, Yi Zhang, Junjie Zhang, Zheng Hao, Xiaofeng Yin

**Affiliations:** Department of Neurosurgery, Second Hospital of Shanxi Medical University, Taiyuan, China

**Keywords:** oncolytic virus, glioma, receptor, immune response, tumor progression

## Abstract

**Background:**

Oncolytic viruses (OVs) achieve selective cytolysis via tumor-specific entry receptor. However, the prevalence of OVs receptors in malignant tumors has not been fully determined yet. Here, we systematically identify and characterize critical cellular entry receptors for clinically relevant OVs, particularly focusing on SCARB2 expression and its potential therapeutic implications for oncolytic Enterovirus A71 (EV-A71) therapy in glioma.

**Methods:**

A systematic literature review was performed to summarize key entry receptors of major oncolytic viruses. Transcriptomic and proteomic data from TCGA, CPTAC, HPA, GEPIA2, CGGA and clinical databases were analyzed to profile receptor expression and clinical relevance across cancer types, especially glioma. Immunofluorescence and RNAi assays in glioblastoma (GBM) cell lines were conducted to assess SCARB2 localization, expression, and cellular functional roles.

**Results:**

Pan-cancer analyses revealed widespread overexpression of key viral receptors. SCARB2 significantly was overexpressed in glioma compared to brain tissues. Elevated SCARB2 protein levels were particularly noted in high-grade gliomas. Further *in vitro* assays confirmed SCARB2 localization primarily at the cell membrane in glioblastoma cells. Additionally, SCARB2 expression correlated with molecular subtype, immune subtype, and tumor-infiltrating lymphocyte composition in gliomas. Functional studies demonstrated that SCARB2 knockdown and EV-A71 infection markedly reduced GBM cell proliferation and enhanced cell apoptosis rate, suggesting its critical role in facilitating viral entry and subsequent antitumor effects.

**Conclusions:**

SCARB2 serves as a critical cellular receptor for EV-A71-mediated oncolytic activity in glioma. Elevated SCARB2 expression in GBM highlights its potential as both a therapeutic target and predictive biomarker for selecting glioma patients responsive to oncolytic EV-A71 therapy.

## Introduction

Oncolytic virus therapy represents an innovative and rapidly advancing therapy in tumor treatment, employing genetically modified or naturally viruses to target and eliminate tumor cells selectively (Bommareddy et al., 2018; Ma et al., 2023). This therapeutic strategy integrates the viruses’ ability of replicating in cancer cells and their capacity to activate anti-tumor immune response (Kalafati et al., 2023; Malhotra and Kim, 2023). Beyond directly causing cell death, these viruses release tumor-specific antigens, which are recognized by the immune system, thereby initiating a robust immune response against the cancer (Roberts et al., 2006; Alwithenani et al., 2025). The approval of oncolytic viruses, T-VEC, have exhibited an excellent safety profile and efficacy following intratumoral administration (Shalhout et al., 2023). It is hypothesized that oncolytic viruses are specific to tumor cells and have a wide range of immune stimulation dimensions (Raja et al., 2018). The use of host-adaptive immune responses results in the extensive impact of oncolytic viruses and sensitively distinguishing tumor cells (Raja et al., 2018).

The key of oncolytic virus therapy is the interaction between the oncolytic virus and specific receptors on the surface of tumor cells (Kuryk and Møller, 2020). The receptors expression on tumor cells determines the ability of the virus to bind, penetrate, and replicate within the target cells (Miles et al., 2017). Extensive research has been conducted to uncover the receptors driving oncolytic virus infection. In particular, the oncolytic virus receptors such as nectin-1, 3-O-sulfated heparan sulfate (Ishino et al., 2021), AXL and integrin αvβ5 (Victorio et al., 2024) were identified during progression of oncolytic virus infection in tumor cells. Understanding the molecular mechanisms of oncolytic virus receptor interaction is essential for improving therapeutic outcomes and minimizing off-target effects.

Glioma is the most frequent form of primary tumors of the brain, arising from glial cells, which play a key role in maintaining the structure and function of the nervous system (Gladson et al., 2010; Reifenberger et al., 2017; Reni et al., 2017). Glioma is categorized into various subtypes based on histopathological features and molecular characteristics, such as less aggressive low-grade glioma (LGG) (e.g. astrocytoma) (Morshed et al., 2019) and highly malignant high-grade gliomas (e.g. glioblastoma multiforme, GBM) (Luo et al., 2020). High-grade gliomas are characterized by rapid growth, extensive invasiveness, and poor prognosis, making them as a significant focus of oncological research (Weller et al., 2024). Until 2021, glioma moved up to become the second most common cancer death before colorectal cancer (Siegel et al., 2024). Despite advancements in surgical resection operation, chemotherapy, and radiotherapy, the five-year survival rate for glioma patients remains dismal, at less than 15% (Chen et al., 2017). The treatment of glioma remains challenging due to its infiltrative growth patterns and molecular heterogeneity (Nicholson and Fine, 2021; Frosina, 2023). Current research is actively exploring innovative therapeutic approaches, including targeted therapies, immunotherapy, and gene therapy, aiming to enhance patient outcomes for glioma (Pulkkanen and Yla-Herttuala, 2005; Huang et al., 2023; Varela et al., 2023). Understanding the molecular mechanisms driving glioma progression is critical for developing more effective and personalized therapies.

Building upon these findings, the present study investigates the role of SCARB2, the receptor for Enterovirus A71, its role in immune regulation, and its impact on tumor progression within glioma. Using bioinformatics and *in vitro* experiments, this research seeks to identify proteins associated with SCARB2 and explores the regulatory pathways involved in glioma, offering important insights into its molecular mechanisms and novel therapeutic approaches for glioma treatment.

## Materials and methods

### Data acquisition and processing

Transcriptomics data and tumor clinical data for the TCGA-GBM and TCGA-LGG cohorts were downloaded from the GDC data portal. After converting the TPM values of TCGA and GTEx to log (TPM + 1), gene expression was analyzed using the R software v2.25.3. For the Chinese brain tumor cohorts, sequencing data analysis was conducted using the CGGA database (https://www.cgga.org.cn/help.jsp#download) (Zhao et al., 2021). Functional enrichment analysis was conducted utilizing data from the Gene Ontology (GO) and the Kyoto Encyclopedia of Genes and Genomes (KEGG) through the DAVID Functional Annotation Bioinformatics Microarray Analysis tool (https://davidbioinformatics.nih.gov/gene2gene_new.jsp). The SCARB2 gene, along with its significantly correlated genes, was employed to construct a protein-protein interaction (PPI) network using the STRING database (https://cn.string-db.org/). Additionally, Gene Set Enrichment Analysis was performed using the software available on the GSEA website (https://www.gsea-msigdb.org/gsea/index.jsp).

### Immunofluorescence microscopy

GBM cells were seeded at a density of 5 × 10^4^ in a 6-well plate and incubated overnight in an environment of 5% CO_2_ at 37 °C. The cells were washed twice with ice-cold phosphate-buffered saline (PBS) after siRNA transfection or virus infection. Subsequently, the cells were fixed with a 4% paraformaldehyde solution for 10 min at room temperature. and permeabilized using a solution containing 0.3% Triton X-100 diluted in PBS. Next, glioma cells were incubated in a blocking buffer consisting of 2% bovine serum albumin (BSA) solution and then were treated with primary antibodies (Rabbit SCARB1 antibody, #90332, Cell Signaling technology, Danvers, USA) at 1:1000 dilution, followed by Goat Anti-Rabbit IgG (FITC Conjugate, #86426, Cell Signaling technology). Nuclear counterstaining was performed using DAPI. Images were captured using a Leica confocal microscope.

### Cell culture, transfection and oncolytic virus Enterovirus A71

U87 and U251 cell lines were obtained from the ATCC cell repository. U87 cells were cultured in MEM medium (Gibco, USA), while U251 cells were cultured in DMEM/F12 medium (Gibco, USA). Both MEM and DMEM/F12 media were supplemented with 10% FBS (Gibco, USA). GBM cell line was maintained in a humidified incubator at 37 °C with 5% CO_2_. All experiments were performed using mycoplasma-free cells. Sangon Biotech (Shanghai, China) constructed the siRNAs for negative control (si-NC) and SCARB2 knockdown (si-SCARB2). Cells were seeded at a confluency of 60-70% overnight and subjected to cell transfection using Lipofectamine 3000 (Invitrogen). An EV-A71 strain was acquired from the Institute of Medical Biology at the Chinese Academy of Medical Sciences. Glioma cells were infected with EV-A71 at different multiplicities of infection (MOIs) for a duration of 4 hours. Culture supernatants were collected a for further examination.

### qRT-PCR

The total RNAs from U87 and U251 cell lines were isolated using TRIzol reagent (Qiagen, Hilden, Germany). The concentration and purity of the isolated RNA were determined by measuring the absorbance at 260/280 nm using a NanoDrop ND-1000 Spectrophotometer (Thermo Fisher). Subsequently, complementary DNA (cDNA) was synthesized from 1 µg of RNA using the PrimeScript RT Reagent Kit (Takara Bio, Mountain View, CA). The cDNA was then subjected to qPCR analysis using the PreMix (SYBR Green) Kit. The primer was listed as SCARB2 Forward primer 5’-CGG CGA AGG AAA CCG AAA C-3’, Reverse primer 5’-TGT AGC CCC AGA GCA ATT CG-3’. The relative expression levels of each gene were normalized to β-actin, and quantified using the 2^−ΔΔCt^ method.

### LDH release and MTT assays

The cytotoxicity index was assessed using a lactate dehydrogenase (LDH) release assay with the LDH Cytotoxic Assay Kit (Abcam). Glioma cells were either transfected with siRNAs or exposed to EV-A71 infection, then seeded into a 96-well plate and grown in MEM medium. The cell culture supernatant was mixed with the LDH reaction mix (Beyotime, Beijing, China) over 30 minutes. The samples were determined at 450 nm using a microplate reader. Assessment of cell proliferation was carried out via the MTT assay (Beyotime). Each well of the 96-well plate was seeded with 1 × 10^4^ cells. At various time intervals, MTT solution (5 mg/mL) was introduced into the wells, which were then incubated at 37 °C for four hours. Once the formazan crystals were solubilized with DMSO, the OD450 was measured at 570 nm using a microplate reader.

### Apoptosis assay

Apoptotic capacity was evaluated through flow cytometry. Cells were obtained, washed with PBS at 4 °C, and treated with FITC-annexin V and propidium iodide solution. After incubation at 37 °C for 30 minutes, apoptotic cell percentages were analyzed by flow cytometry and processed using FlowJo software.

### Statistical analysis

Statistical analysis was performed using SPSS software, version 26.0. Results are expressed as the mean with standard deviation (SD). For comparing differences between two experimental groups, an unpaired two-tailed Student’s t-test was employed. A p-value of less than 0.05 was set as statistical significance.

## Results

### Systematic review of oncolytic virus entry receptors and their associated tumor therapy

To establish a comprehensive framework for evaluating potential oncolytic virotherapies, we conducted a systematic literature review to identify the primary cellular entry receptors for a panel of leading oncolytic viruses. The objective was to survey the current understanding of the molecular gateways these viruses use for cell entry and to map their established or preclinical efficacy across various human malignancies. The results of this systematic survey are summarized in **Table 1**. This table details each oncolytic virus, the cancer types in which its oncolytic activity has been reported, its key cellular receptors, and the corresponding PubMed references (PMIDs) that substantiate these findings. The selected references provide evidence for both the receptor-virus interaction and the therapeutic application of the virus in specific cancer models or clinical settings. We focused on several key receptors known to facilitate oncolytic virus entry, including HVEM and NECTIN1 receptors for oncolytic herpes simplex virus, SCARB2 and SELPLG receptors for oncolytic Enterovirus A71, AXL receptor for oncolytic Zika Virus and Measles virus, CD46 receptor for Adenovirus and Measles Virus and LDLR receptor for Vesicular stomatitis virus.

**Table 1:** Cellular receptors, targeted malignancies, and supporting literature for key oncolytic viruses.

Oncolytic virus	Targeted cancer types	Cellular entry receptors	Key references
Herpes Simplex Virus (HSV)	Melanoma, Sarcoma, Glioblastoma, Lymphoma	HVEM, Nectin-1	PMID: 33738338 PMID: 19351838 PMID: 35384530
Enterovirus A71 (EV-A71)	Malignant Glioma	SCARB2, PSGL-1 (SELPLG)	PMID: 32304669 PMID: 31924205 PMID: 36992493
Zika Virus (ZIKV)	Glioblastoma Multiforme (GBM), Medulloblastoma, Ependymoma	AXL, Integrin αvβ5	PMID: 31956038 PMID: 38308299
Adenovirus	Ovarian Cancer, Bladder Cancer	CD46	PMID: 31362478 PMID: 30201920 PMID: 33147799
Measles Virus	Ovarian Cancer, Glioblastoma	CD46, SLAMF4 (Nectin-4)	PMID: 25398436 PMID: 38216554 PMID: 27782084
Vesicular Stomatitis Virus (VSV)	Colorectal Cancer, Pancreatic Cancer, Lung Cancer	LDLR, LDLR Family (e.g., VLDLR)	PMID: 31731579 PMID: 33903244 PMID: 24246772

### Pan-cancer profiling of oncolytic virus receptor expression reveals tumor-specific alterations

We firstly analyzed transcriptomic data from TCGA. Our analysis revealed that the mRNAs encoding these six critical oncolytic viruses entry receptors were frequently and significantly upregulated in a majority of the 33 cancer types compared to normal tissues (**Supplementary Figure S1**). Specifically, HVEM(*tnfrsf14*)mRNA, a primary receptor for oncolytic Enterovirus A71, was broadly overexpressed in GBM (**Supplementary Figure S1**; p < 0.01). NECTIN1, a primary receptor for oncolytic herpes simplex virus, was broadly overexpressed, with particularly high levels observed in Lung Squamous Cell Carcinoma (LUSC) and Cervical Cancer melanoma (CESC). HVEM and CD46 also demonstrated significant upregulation patterns across various epithelial and mesenchymal tumors (**Supplementary Figure S1**). In contrast, the expression of SELPLG was not significantly different in tumors and normal tissues, and in some tumors, it was lower than in normal tissues (**Supplementary Figure S1**). The expression of AXL and LDLR was not significantly different in glioma compared to normal tissues (**Supplementary Figure S1**).

### Focused analysis of SCARB2 protein expression in glioma

**Figure 1:**
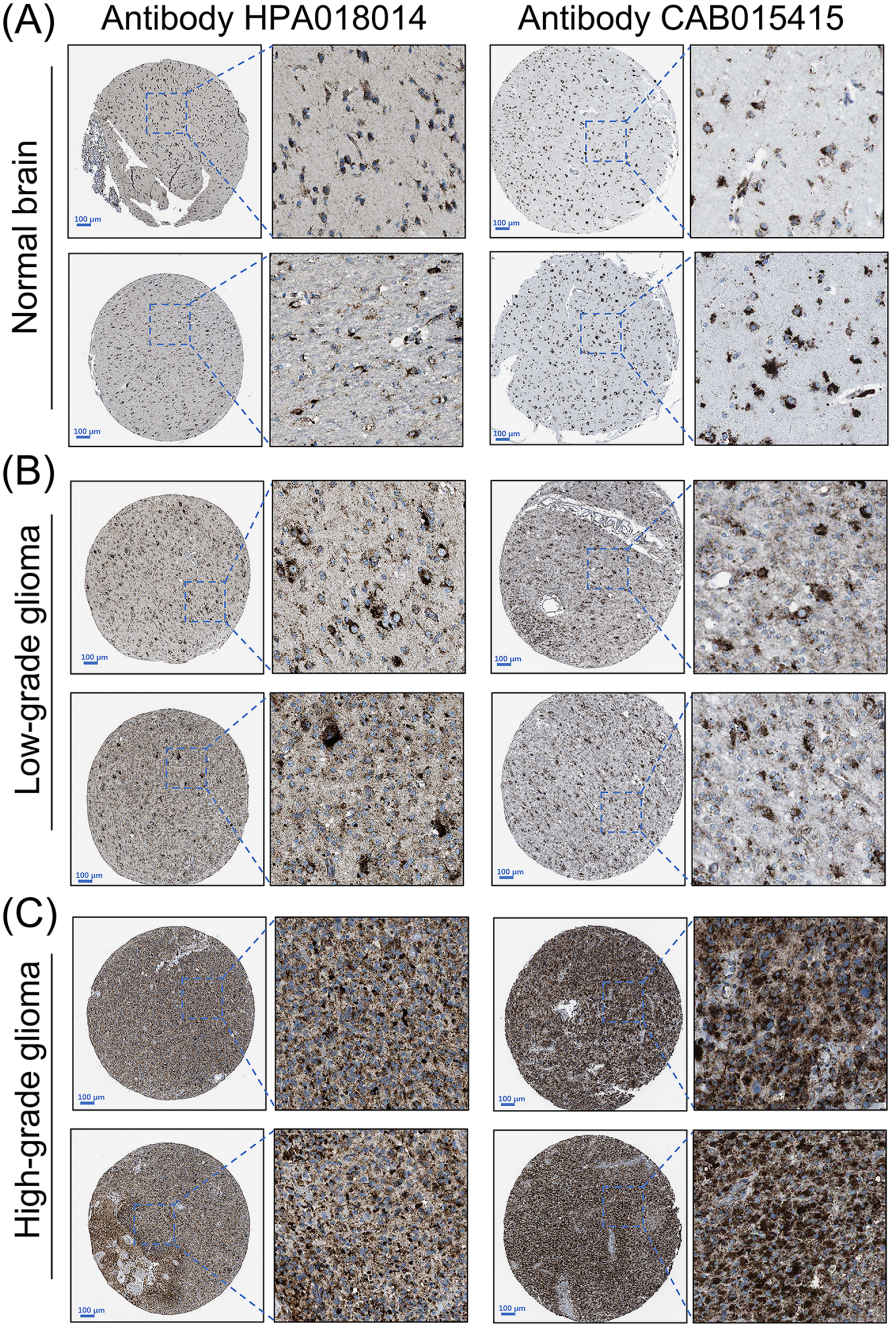
Protein expression levels of SCARB2 in glioma tissues. Representative immunohistochemistry (IHC) images of **(A)** normal brain, **(B)** low-grade glioma and **(C)** high-grade glioma tissues stained for SCARB2. Scale bar: 100 µm.

**Figure 2:**
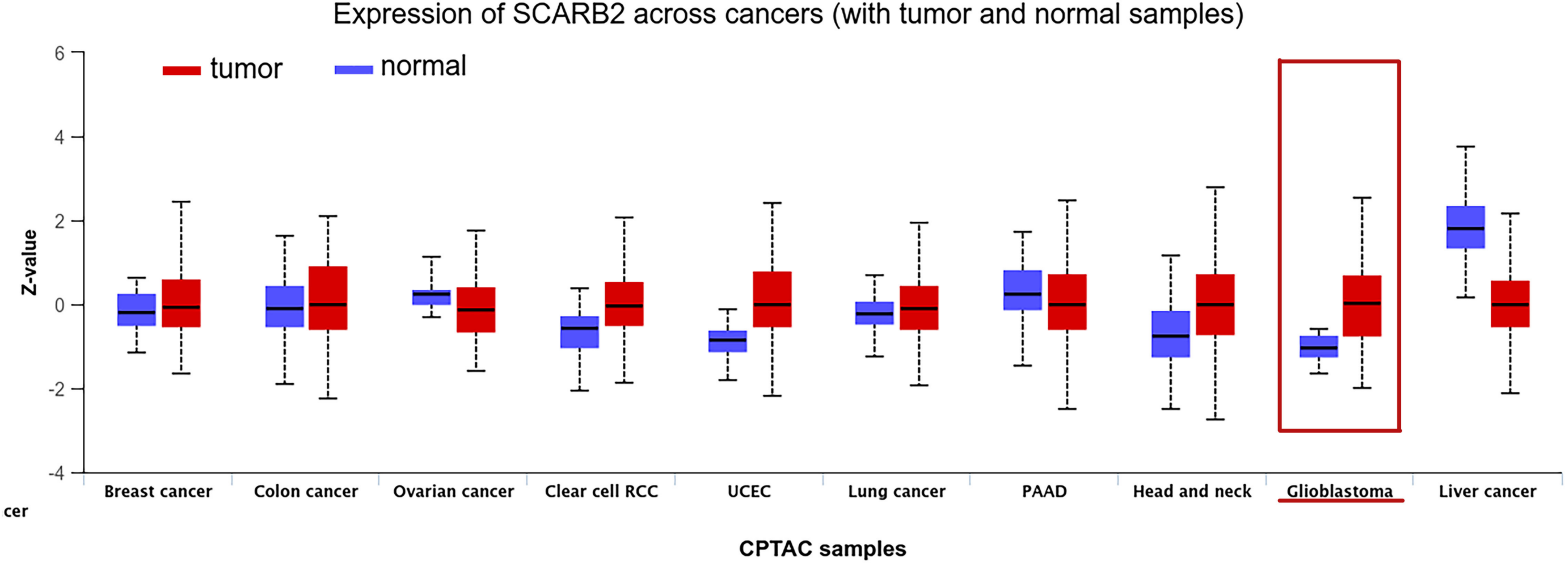
Protein expression levels of SCARB2 in CPTAC samples across cancers.

Our pan-cancer screening identified the gene SCARB2 as a subject for the focused investigation in glioma. To independently validate the protein expression of SCARB2, we analyzed the immunohistochemistry (IHC) data from the Human Protein Atlas (HPA). The IHC image confirmed a clear difference in SCARB2 protein levels between high-grade glioma and LGG. SCARB2 protein was detected at low to negligible levels in LGG compared with normal brain tissues (**Figures 1A, B**). In contrast, a substantial proportion of high-grade glioma samples exhibited moderate to high-intensity staining for SCARB2 (**Figure 1C**). The observed staining pattern was primarily cytoplasmic with granular features, consistent with the protein’s known localization to the endo-lysosomal pathway. Moreover, the proteomics data from CPTAC database confirmed that SCARB2 protein expression was substantially increased in GBM relative to normal samples (**Figure 2**). This analysis of an independent dataset supports our initial transcriptomic findings and establishes increased SCARB2 protein abundance in glioma.

### Analysis of SCARB2 mRNA expression and its clinical relevance in glioma

**Figure 3:**
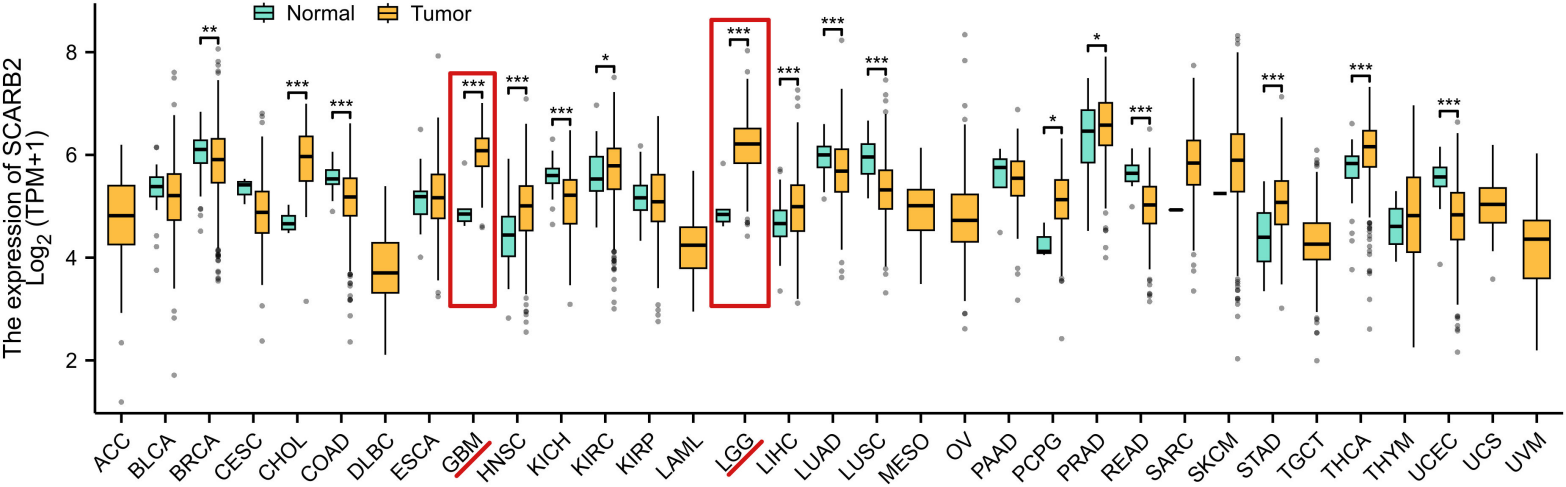
mRNA expression levels of SCARB2 in pan-cancer. * p < 0.05, ** p < 0.01, *** p <0.001.

**Figure 4:**
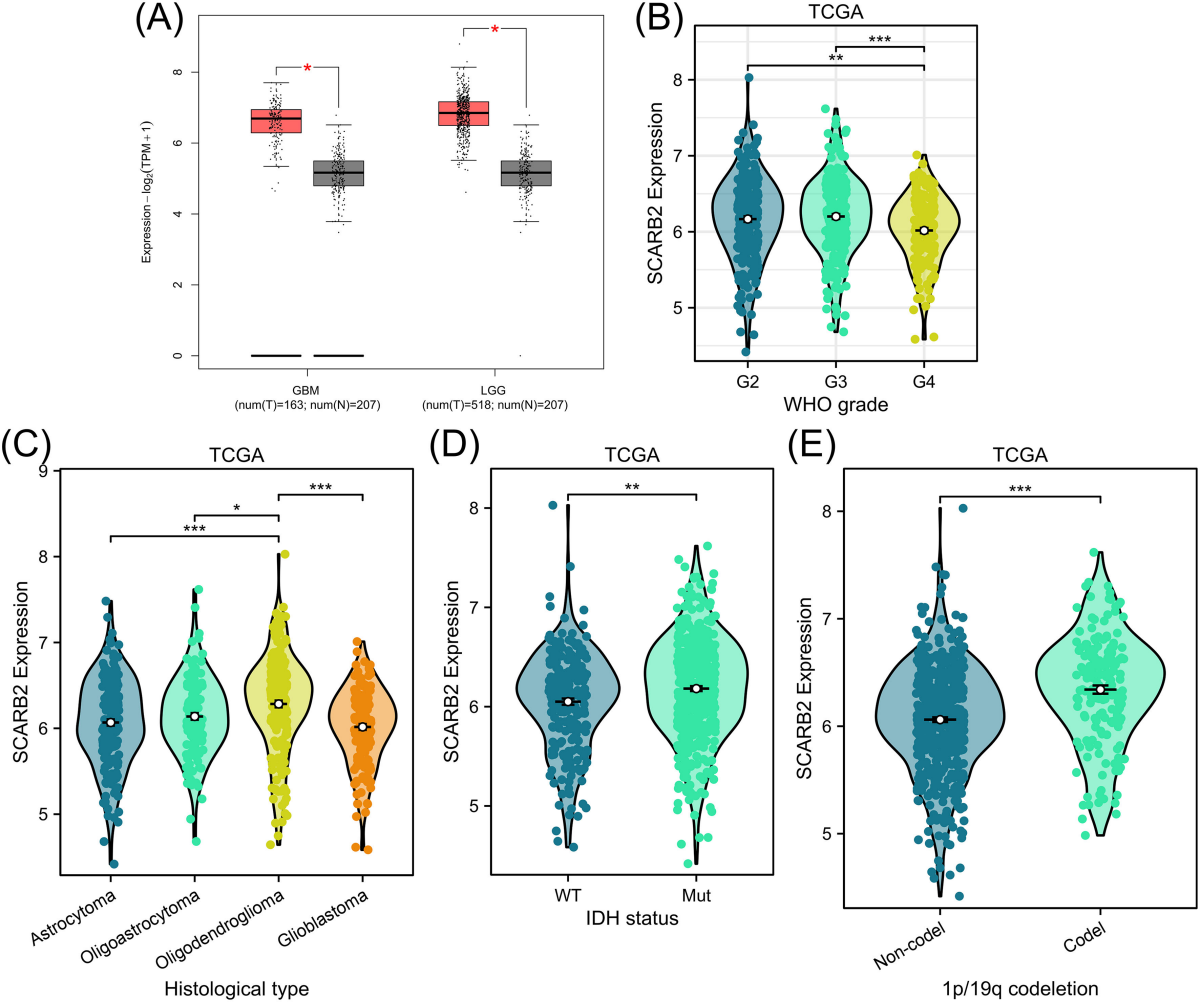
Analysis of SCARB2 mRNA expression and its clinical relevance in TCGA-glioma cohorts. **(A)** Box plots were derived from GEPIA database based on TCGA-LGG/GBM cohorts and GTEx data. **(B)** A mRNA expression analysis of SCARB2 level in different WHO grades of glioma patients. **(C)** A mRNA expression analysis of SCARB2 level in different histological types of glioma patients. **(D, E)** SCARB2 expression analysis in glioma with different IDH status and 1p/19q codeletion. *p < 0.05, **p < 0.01, ***p < 0.001. .

**Figure 5:**
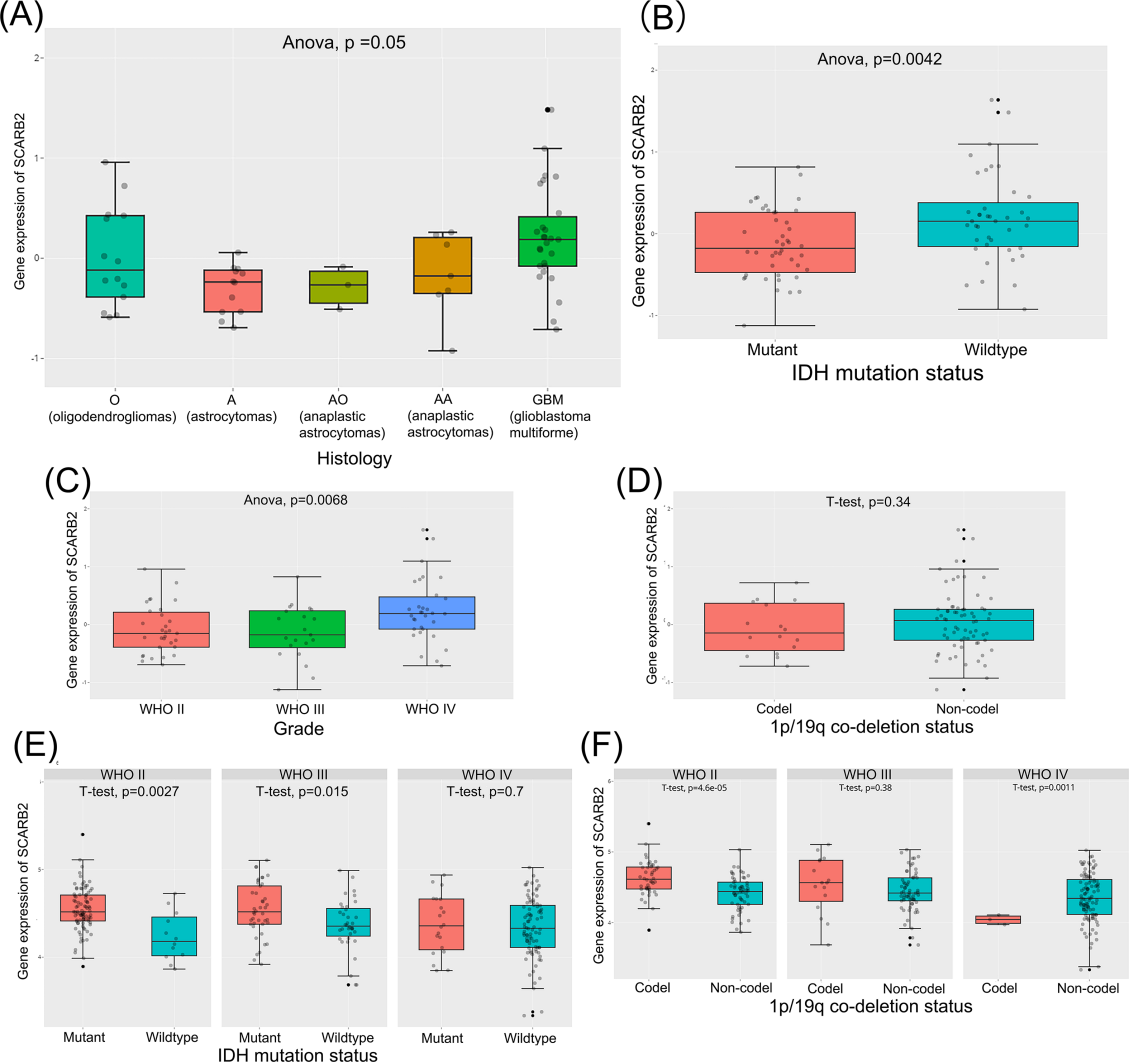
Analysis of SCARB2 mRNA expression and its clinical relevance in CGGA-glioma cohorts. **(A, C)** A mRNA expression analysis of SCARB2 level in different histological types and histological types of glioma patients. **(B, D–F)** SCARB2 expression analysis in glioma with different IDH status and 1p/19q codeletion.

To explore potential role of SCARB2 in glioma progression, we analyzed mRNA expression data for SCARB2 across various cancer tissues from 33 cancer types or subtypes in the TCGA database. The expression levels of SCARB2 were significantly higher in the cancer tissues of GBM and LGG compared to the corresponding normal tissues (**Figure 3**). To validate the clinical significance of SCARB2 expression in glioma, we utilized the GEPIA2 database to analyze glioma tissue microarrays. SCARB2 exhibited higher expression levels in glioma tissues (both GBM and LGG) in comparison with brain tissues (**Figure 4A**; p < 0.05). Interestingly, inconsistent with the IHC protein staining results, patients with glioma with high WHO grade and histological type (GBM) showed a decreased SCARB2 mRNA expression (**Figures 4B, C**; p < 0.05, p < 0.01, p < 0.001). SCARB2 mRNA expression was highly expressed in the glioma cases with IDH mutation and 1p/19q codeletion (**Figures 4D, E**; p < 0.001). Using the CGGA dataset, we examined SCARB2 expression in 2,000 clinical brain tumor samples from Chinese patient cohorts. Notably, SCARB2 expression levels were higher in glioma with advanced tumor stages (**Figures 5A, C**; p < 0.05). Furthermore, elevated SCARB2 expression was particularly evident in patients with IDH wildtype status (**Figure 5B**; p < 0.01), except for WHO grade II and III gliomas (**Figure 5E**; p < 0.05, p < 0.01). However, no statistically significant differences in SCARB2 expression were observed between the 1p/19q co-deletion and non-co-deletion groups across all gliomas (**Figure 5D**). Interestingly, SCARB2 expression in WHO grade II and IV gliomas was found to correlate with 1p/19q co-deletion status (**Figure 5F**; p < 0.01, p < 0.001).

### GO enrichment annotation analysis, KEGG enrichment pathway analysis and GSEA analysis

**Figure 6:**
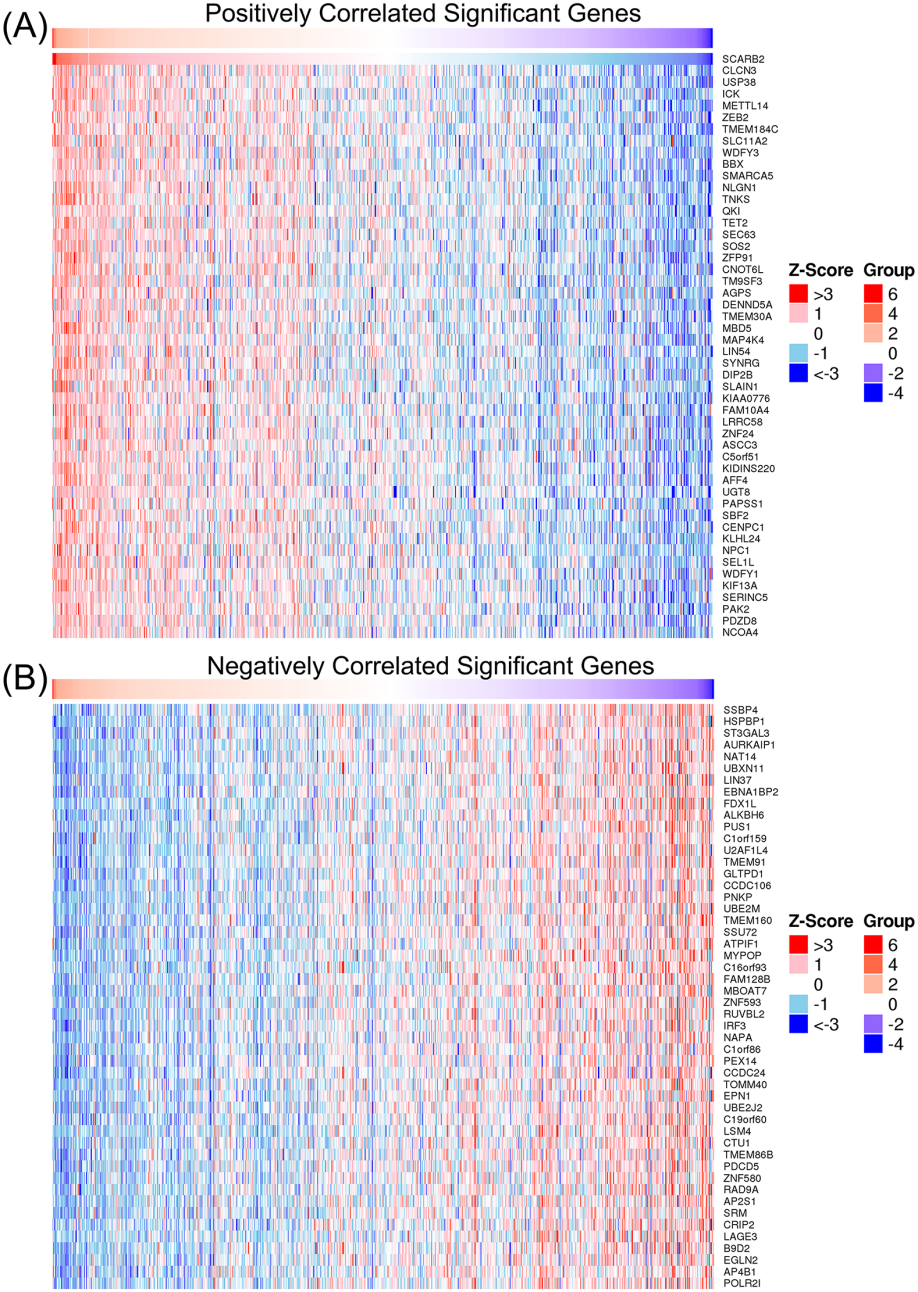
Heap map of genes significantly correlated with SCARB2. **(A)** The top 50 genes positively correlated with SCARB2. **(B)** The top 50 genes negatively correlated with SCARB2.

**Figure 7:**
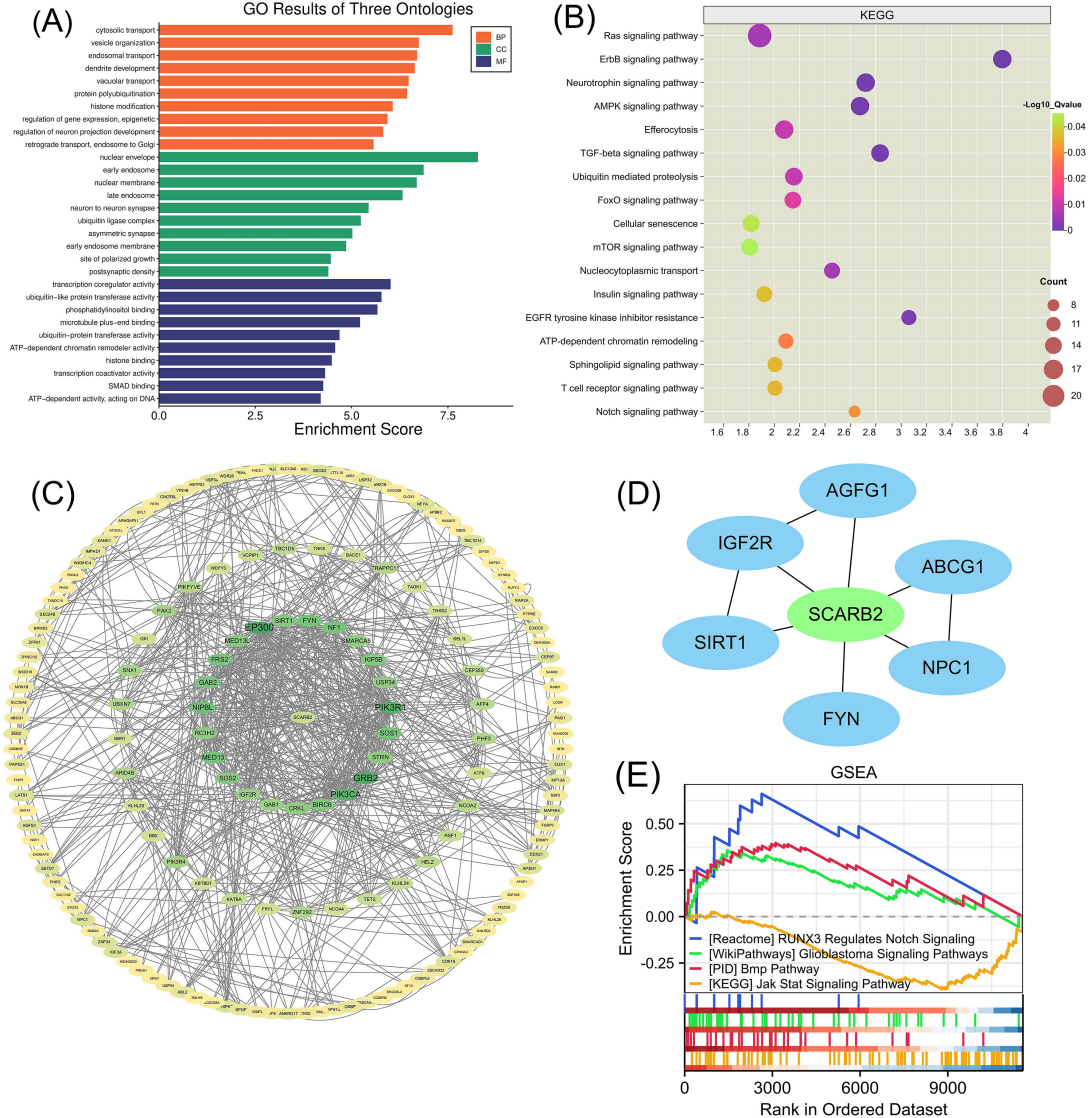
GO enrichment annotation analysis, KEGG enrichment pathway analysis and GSEA analysis. **(A)** Gene Ontology analysis and **(B)** KEGG pathway analysis of SCARB2-related proteins in glioma tissues. **(C)** Graphical representation of the positively significant correlated proteins and PPI network. **(D)** Construction of a PPI network containing SCARB2 and most correlated proteins using CytoHubba application. **(E)** GSEA with TCGA-glioma RNA-seq dataset disclosed a significant enrichment of signaling pathway in glioma patients with high SCARB2 expression.

To investigate the molecular patterns of gene expression associated with SCARB2 expression and to identify which downstream signaling pathways are activated upon SCARB2 expression, we performed the association analysis between SCARB2 and other genes in TCGA-GBMLGG by using LinkedOmics (Vasaikar et al., 2017). Heat map showed the top 50 positively and negatively correlated significant gens with SCARB2 expression in glioma (**Figure 6**). Using gene ontology (GO) analysis, we found that these genes positively correlated with SCARB2 were enriched in processes such as cytosolic transport, nuclear envelope, transcription coregulator activity and ubiquitin-like protein transferase activity (**Figure 7A**). Further KEGG enrichment analysis revealed that several cell proliferation and cell apoptosis processes, such as Ras signaling pathway, AMPK signaling pathway, mTOR signaling pathway and Notch signaling pathway, were activated in the top 50 positively correlated significant gens (**Figure 7B**). A PPI network was constructed for the positively significant genes correlated with SCARB2 using the STRING database and Cytoscape software. **Figure 7C** show the PPI network consisting of the 167 gene nodes and 770 edges correlated with SCARB2 (R > 0.4, confidence more than 0.4). Furthermore, we utilized the CytoHubba application to construct particular PPI network containing SCARB2, IGF2R, SIRT1, FYN, NPC1, ABCG1 and AGFG1 nodes (**Figure 7D**). GSEA consistently demonstrated the significant enrichment of RUNX3/Notch signaling, BMP pathway and GBM signaling pathway in group with high SCARB2 expression levels (**Figure 7E**).

### SCARB2 expression was correlated with tumor-infiltrating lymphocytes in glioma

**Figure 8:**
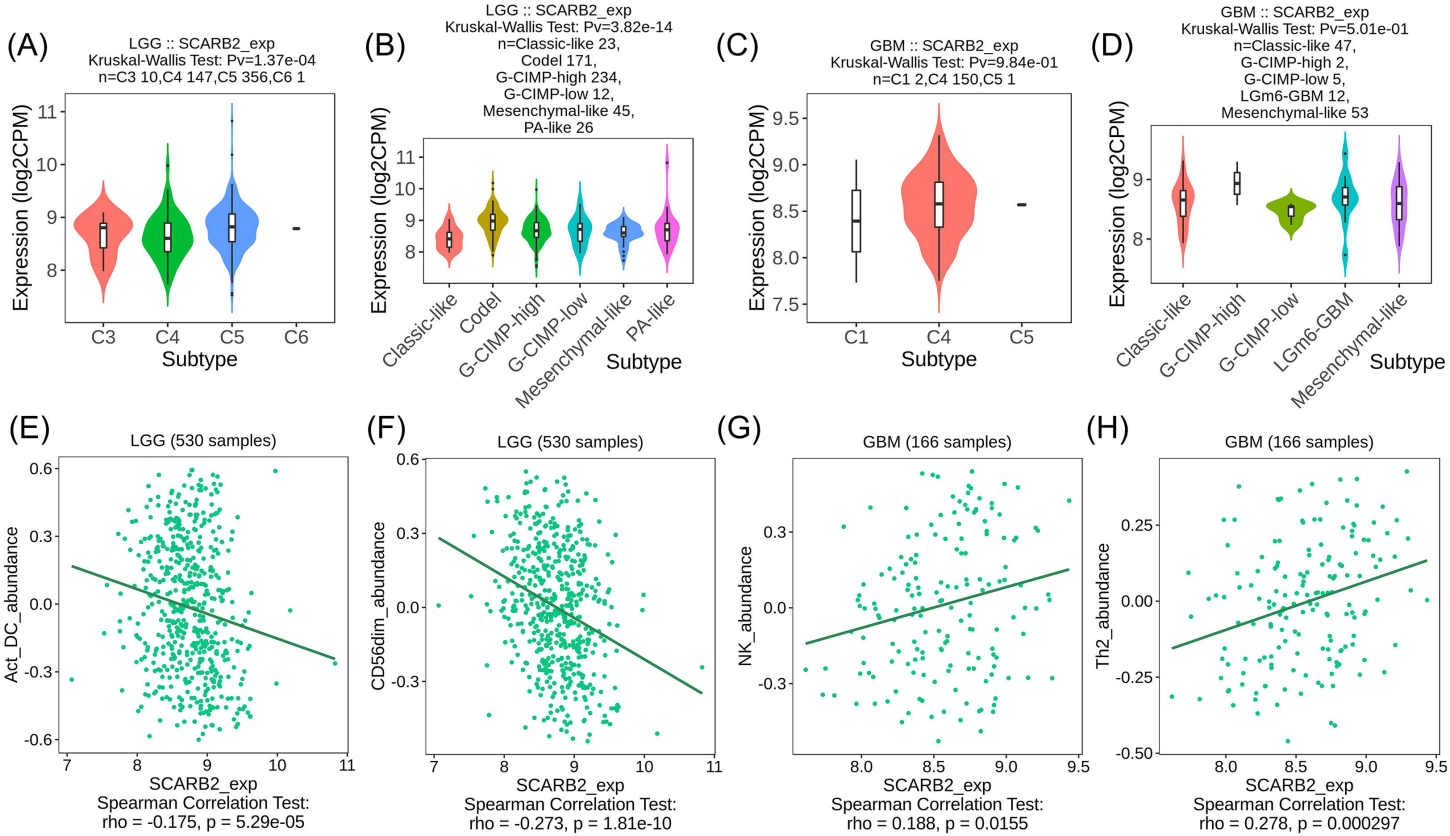
SCARB2 expression was correlated with TILs in LGG. **(A, C)** Analysis of SCARB2 expression levels in different immune subtypes of LGG and GBM. **(B, D)** Analysis of SCARB2 expression levels in different molecular subtypes in LGG and GBM. **(E–H)** Correlation between SCARB expression and TILs in LGG and GBM. C1: wound healing, C2: IFN-gamma dominant, C3: inflammatory, C4: lymphocyte depleted, C5: immunologically quiet, C6: TGF-b dominant.

To explore the interactions between tumor and immune system, we utilized the TISIDB database to investigate the distribution of SCARB2 expression across immune and molecular subtypes in glioma. Specifically, SCARB2 expression was correlated with immune subtypes (C3 inflammatory, C4 lymphocyte depleted and C5 immunologically quiet) (**Figure 8A**) in LGG but not related to any immune subtypes in GBM (**Figure 8C**). Additionally, in glioma, excluding GBM, various molecular subtypes showed a significant correlation with levels of SCARB2 expression (**Figure 8B, D**). As TILs are promising components in immunotherapy for cancers, we further explored the correlation between abundance of TILs and SCARB2 level in LGG and GBM. SCARB2 levels were negatively correlated with infiltrating levels of active dendritic cells (rho = -0.175) and CD56dim natural killer cells (rho = -0.273) in LGG (**Figures 8E, F**). In addition, SCARB2 expression levels were positively related to infiltrating levels of natural killer cells (rho = 0.188) and Th2 macrophages (rho = 0.278) in GBM ((**Figure 8G, H**).

### SCARB2 is localized in the membrane U87 cells

**Figure 9:**
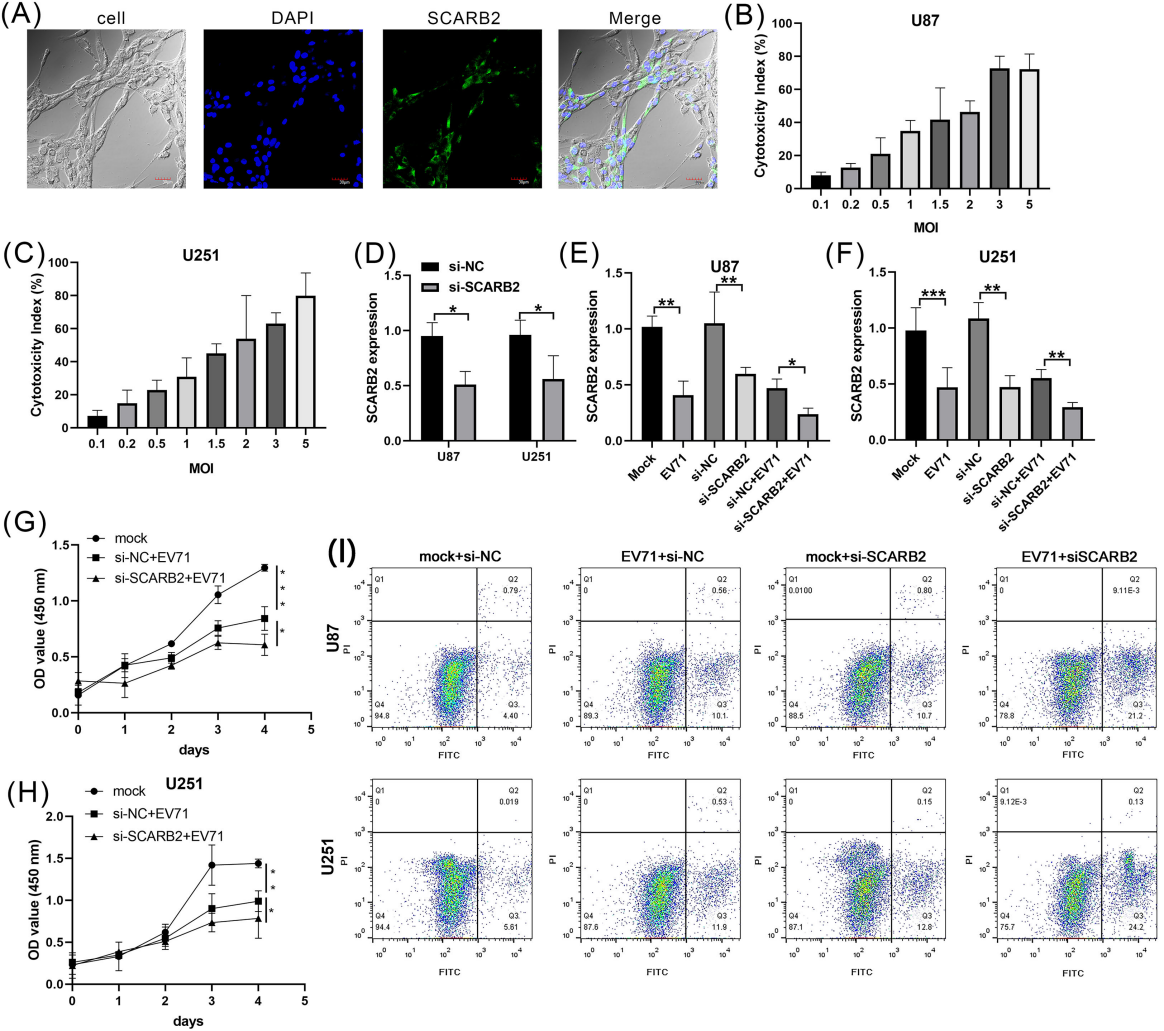
Oncolytic virus EVA71 inhibited glioblastoma cell proliferation via regulating SCARB2. **(A)** Localization of SCARB2 in U87 cells. Cells were fixed and stained with a SCARB2-specific antibody and a FITC-conjugated secondary antibody (green), revealing a strong signal at the cell surface. The nucleus was counterstained with DAPI (blue). Scale bar = 30 µm. **(B, C)** GBM cell lines, U87 and U251, were infected with EVA71 at different MOIs for 48 h and the cytotoxicity was assessed by LDH assay. **(D)** GBM cell lines were transfected with si-NC or si-SCARB2, then SCARB2 expression was detected with qPCR. **(E, F)** GBM cells transfected with si-NC or si-SCARB2 were infected with EVA71 (MOI = 1.5), and then SCARB2 expression was detected with qPCR. **(G, H)** GBM cells transfected with si-NC or si-SCARB2 were infected with EVA71 (MOI = 1.5), and then cell proliferation was detected with MTT assay. *p < 0.05, **p < 0.01, ***p < 0.001. **(I)** Apoptotic rates in the indicated GBM cells that were infected with EV71 or transfected with siRNAs.

To assess the expression and subcellular localization of SCARB2 in GBM cells, we performed immunofluorescence staining on the human U87 cell line. The cells were stained for endogenous SCARB2, which was visualized using a FITC-conjugated secondary antibody (green), and nuclei were counterstained with DAPI (blue). Confocal microscopy analysis revealed that SCARB2 is robustly expressed in U87 cells (**Figure 9A**). The protein displayed a distinct localization pattern that was predominantly localized to the cell periphery, clearly outlining the membrane of the cells. While some diffuse signal was occasionally noted in the cytoplasm, the most consistent staining was observed at the cell surface. This membrane-specific localization pattern aligned with prior reports demonstrating SCARB2’s function as a cell surface receptor for enterovirus internalization. Very weak SCARB2 signal was detected within the DAPI-stained nuclei.

### Oncolytic virus EV-A71 inhibited GBM cell proliferation via regulating SCARB2

To detect the oncolysis role of EV-A71 in glioma cells, we firstly assess the oncolytic potential of EV-A71 in U87 and U251 cell lines. The GBM cells exhibited a dose-dependent increase in cytotoxicity upon EV-A71 infection (**Figures 9B, C**). The infection with EV-A71 at a multiplicity of infection (MOI) of 1.5 results in a 50% reduction in the viability. The MOI of 1.5 showed the appropriate ability to kill the GBM cells, so we selected MOI = 1.5 as the candidate dose for further study. To also test the role of SCARB2 receptor in GBM cells, we co-cultured control (si-NC) or SCARB2 knockdown (si-SCARB2) with U87 or U251 cells. qPCR analysis further confirmed that SCARB2 knockdown markedly decreased SCARB2 mRNA level in both U87 and U251 cells (**Figure 9D**; p < 0.05). In GBM cells, which expresses high endogenous levels of SCARB2, EV-A71 infection or SCARB2 knockdown significantly reduced the SCARB2 expression levels (**Figures 9E, F**; p < 0.05, p < 0.01). Furthermore, due to the knockdown of SCARB2 in glioma cells, EV-A71 infection further inhibited SCARB2 expression (**Figure 9E, F**; p < 0.05, p < 0.01). As revealed by the MTT assays, EV-A71 infection led to inhibited cell growth of GBM cell line, while SCARB2 knockdown further inhibited the malignant features (**Figure 9G, H** p < 0.05, p < 0.01, p < 0.001). Flow cytometry assays using FITC-annexin V and PI staining revealed an elevated rate of apoptosis in both EV-A71-infected U87 and U251 cells (**Figure 9I**). This pro-apoptotic effect was also shown in the si-SCARB2 group (**Figure 9I**). Combination treatments of EV infection and si-SCARB2 markedly enhanced cell apoptosis in glioma cells compared to monotherapies **(Figure 9I**).

## Discussion

Oncolytic viruses are used as therapeutic agents to battle against tumor cells and activate the immune system (Lecoultre et al., 2024). In recent years, significant advancements have been made in our understanding of oncolytic viruses and its potential implications on clinical treatment. However, the precise intracellular mechanisms through which oncolytic viruses contribute to cancer immunotherapy remain to be thoroughly elucidated. In our study, we demonstrate that the oncolytic virus EV-A71 receptor is overexpressed in LGG and GBM tissues. The absence of SCARB2 leads to a substantial reduction in the capacity for glioma cell growth and migration within *in vitro* models. This research uncovers a previously unidentified mechanism involving SCARB2, which appears to hold a fundamental role in the process of glioma carcinogenesis.

The efficacy of oncolytic virotherapy is fundamentally dependent on the virus’s ability to selectively enter and replicate within tumor cells (Yamaguchi and Uchida, 2018). This process is initiated by the binding of viral glycoproteins to specific receptors on the cell surface (Tian et al., 2022). A thorough understanding of these virus-receptor interactions and the expression patterns of these receptors across different cancer types is therefore essential for predicting viral tropism and guiding the rational design of clinical trials. Our investigation systematically surveyed the PubMed database to collate the primary entry receptors for a panel of promising oncolytic viruses and to identify the full spectrum of malignancies in which they have demonstrated therapeutic potential. The solid findings of extensive literature review are presented in our research. The **Table 1** outlines each oncolytic virus, the range of cancer types it has been reported to effectively target, its principal cellular entry receptors, and the corresponding peer-reviewed PubMed references that substantiate these findings. Each virus entry is supported by multiple citations to ensure comprehensive validation of both the receptor mechanisms and the therapeutic applications in specific cancers.

Oncolytic virus therapy has been reported as a novel option in the treatment of malignant gliomas. Recently, studies have indicated a correlation between glioma treatment and oncolytic viruses (Rius-Rocabert et al., 2020), including Zika virus (Li et al., 2022), herpes simplex virus (Hua and Wakimoto, 2019) and adenovirus (Lang et al., 2018). Herpes simplex virus G47Δ-mIL12 has been noted in inducing anti-tumor immunity and the triple combination of G47Δ-mIL12, anti-PD-1 and anti-CTLA-4 extended survival of a GBM mouse model (Saha et al., 2017). Enterovirus is well-known for its causing infectious diseases in infants and young children, as it plays a role in spreading from initial replication sites to the central nervous system, decreasing microvascular endothelial cell viability and inducing apoptosis (Liang et al., 2004; Xie et al., 2024). Recently, patients with colorectal cancer (CRC) indicates that EV-A71 is associated with immune-based anticancer therapy as EV-A71 inhibits tumor growth in nude mice CRC model by repressing Bcl-2 expression (Ruan et al., 2023). In this study, our focus was primarily on exploring the anti-tumor function of oncolytic virus EV-A71.

Previous studies have linked elevated SCARB2 to tumor progression in breast cancer (Zhao et al., 2025) and liver cancer (Wang et al., 2023), where it regulates key pathways, such as MYC acetylation and tumor-infiltrating immune cell levels. Notably, SCARB2 has also been shown as an EV-A71 receptor to modulate EV-A71 cytolytic activity in glioma (Zhang et al., 2020). The exact mechanism by which it influences glioma is not well understood, making it an attractive target for further investigation. Our study demonstrated that SCARB2 levels were upregulated in glioma tissues and associated with glioma WHO grade. In addition, there is significant interaction between SCARB2 and immune subtype within the LGG tumor microenvironment. Hence, elevated SCARB2 levels were associated with immune cell infiltration, indicating the possibility of SCARB2 as an innovative immunotherapy target. In this study, through LDH release and MTT assays, we demonstrated that a MOI of 1.5 oncolytic virus EV-A71 has moderate cytotoxicity on GBM cells and EV-A71 infection inhibited GBM cell proliferation via regulating SCARB2 expression. Moreover, based on the analysis of significantly correlated genes, we propose several mechanisms that may collectively contribute to glioma cell biology. SCARB2 may coordinate with positively correlated significant genes, like IGF2R (Noh et al., 2024), AGFG1, SIRT1 (Liang et al., 2023), ABCG1 (Chen et al., 2016), FYN (Guo et al., 2019) and NPC1, as identified in several literatures. We observed that SCARB2 and positively correlated genes influenced the RUNX3/Notch signaling and BMP pathway in glioma. Our data provided compelling evidence that SCARB2, as EV-A71 receptor in glioma cells, regulates downstream gene expression, offering new insights into the oncolytic virus therapy of tumor progression.

The communication between the tumor and the immune system significantly influences cancer initiation, progression, and therapeutic approaches (Thorsson et al., 2018). In this study, we utilized TISIDB database to investigate tumor and immune system interaction (Ru et al., 2019). C3 subtype (inflammatory) is enriched in SCARB2 expressed LGG cells with the elevated Th17 and Th1 genes and tumor cell proliferation. In addition, C4 subtype (lymphocyte depleted) was enriched in GBM, and displayed a more significant macrophage signature. Our data also revealed that both of Th2 cytokine signature and SCARB2 expression in GBM patients fosters a tumor-promoting environment and might facilitates tumor growth.

In conclusion, our study highlights the key role of EV-A71 as an oncolytic modulator in glioma, shedding light on its molecular mechanism. We found that SCARB2 expression was significantly upregulated in GBM cells, where it directly interacted with the oncolytic virus EV-A71. This interaction induced the oncolytic activity of EV-A71 in glioma, resulting in suppressed proliferation of glioma cells. These results highlight the importance of SCARB2 and EV-A71 in targeting glioma. Furthermore, SCARB2 and related genes significantly regulated glioma RUNX3/Notch and BMP signaling pathways Overall, our findings reveal SCARB2 as a key receptor for the oncolytic virus EV-A71, suggesting its promise as a therapeutic target in glioma.

## Data Availability

The original contributions presented in the study are included in the article/**Supplementary Material**. Further inquiries can be directed to the corresponding author.

## Supplementary material

Supplementary Figure 1 mRNA expression levels of oncolytic virus receptor in pan-cancer.
