# Supplementary figures and images for: Selective targeting of glioma via the SCARB2 receptor: transcriptomic, proteomic and in vitro functional validation for Enterovirus A71 virotherapy
Source: Front Cell Infect Microbiol. 2025 Oct 23;15:1709002. doi: 10.3389/fcimb.2025.1709002 (PMC12588948; doi:10.3389/fcimb.2025.1709002)

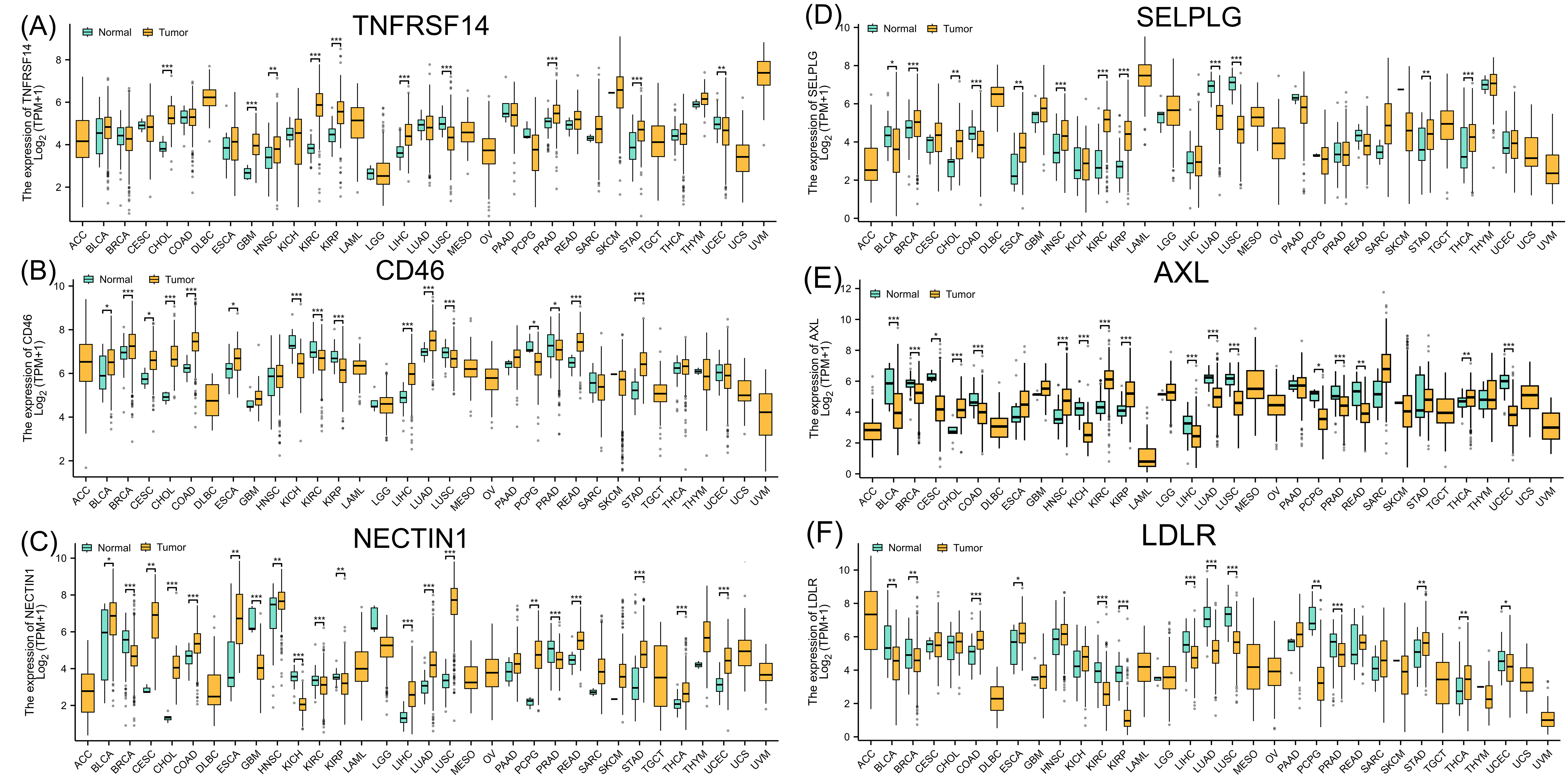

Supplement: Supplementary Figure 1 — mRNA expression levels of oncolytic virus receptor in pan-cancer. [file Image1.tif]
